# Rivalry Onset in and around the Fovea: The Role of Visual Field Location and Eye Dominance on Perceptual Dominance Bias

**DOI:** 10.3390/vision3040051

**Published:** 2019-09-30

**Authors:** Jody Stanley, Jason D. Forte, Olivia Carter

**Affiliations:** Melbourne School of Psychological Sciences, The University of Melbourne, Victoria 3010, Australia; jdf@unimelb.edu.au (J.D.F.); ocarter@unimelb.edu.au (O.C.)

**Keywords:** binocular rivalry, onset rivalry, bistable vision, perceptual bias

## Abstract

When dissimilar images are presented to each eye, the images will alternate every few seconds in a phenomenon known as binocular rivalry. Recent research has found evidence of a bias towards one image at the initial ‘onset’ period of rivalry that varies across the peripheral visual field. To determine the role that visual field location plays in and around the fovea at onset, trained observers were presented small orthogonal achromatic grating patches at various locations across the central 3° of visual space for 1-s and 60-s intervals. Results reveal stronger bias at onset than during continuous rivalry, and evidence of temporal hemifield dominance across observers, however, the nature of the hemifield effects differed between individuals and interacted with overall eye dominance. Despite using small grating patches, a high proportion of mixed percept was still reported, with more mixed percept at onset along the vertical midline, in general, and in increasing proportions with eccentricity in the lateral hemifields. Results show that even within the foveal range, onset rivalry bias varies across visual space, and differs in degree and sensitivity to biases in average dominance over continuous viewing.

## 1. Introduction

When each eye is simultaneously presented with a different image, conscious perception will switch every few seconds between the two possible images, in a phenomenon called binocular rivalry [1,2]. Because an observer’s conscious experience is constantly changing during the viewing of static stimuli, binocular rivalry has been an important tool for understanding the factors that determine the content of conscious visual awareness. One hallmark of the perceptual switching that is experienced while observing rivaling stimuli is that it is unpredictable which image will be perceived at a given time. Recent research has shown, however, that the initial conscious percept in rivalry is not random and that there is generally a bias towards a given image at this first stage [3,4,5,6,7].

The distinction between the characteristics of initial and subsequent viewing periods is relevant for understanding how information from the two eyes is combined to form a conscious visual experience under natural viewing conditions. There is, however, somewhat limited data on what factors affect the onset stage compared to ongoing rivalry, and these studies have had seemingly contradictory results in identifying correlations between onset bias and any bias in average dominance seen over sustained viewing [3,7,8,9]. However, there are some stimulus factors that have been found to affect the likelihood of one image being dominant in consciousness both at onset and during sustained rivalry [5,6,10], including eye of presentation [4,7,9,11,12]. The clear role of eye of presentation at the onset of rivalry demonstrates that eye dominance is highly influential in the onset bias. In fact, rivalry is often used as a test of general eye dominance itself, often termed ‘sensory eye dominance’ [13].

Tests of sensory eye dominance are typically conducted at fixation. However, the strength of the signal from each eye can vary across the visual field. Differences in rivalry switch rate and spatial frequency sensitivity have been shown between the left and right visual fields [14,15]. In addition to hemifields divided along the vertical and horizontal meridian of the visual field, differences can also be detected based on the whether a stimulus falls on the temporal retina (corresponding to the central visual field) or on the nasal retina (corresponding to the lateral portions of the visual field). In normal (non-rivalrous) vision, for example, psychophysical studies using flicker and grating detection to demonstrate asymmetry in sensitivity to stimuli projected onto the temporal and nasal retina, with nasal retina (corresponding to the temporal visual field) being more sensitive. In these cases, the most substantial asymmetry is seen at eccentricities above 20° and some asymmetry at 8°, but no difference at 4° [16]. Similarly, Silva et al. (2008) found a disadvantage in contrast sensitivity for the nasal hemifield, which was also modulated by left/right and dorso/ventral asymmetries [17]. In line with these perceptual differences, anatomical studies show that in the far periphery (greater than 20° eccentricity), the nasal retina (temporal hemifield) has 40% to 50% higher rod and cone density [18] and three times higher retinal ganglion density [19]. Such psychophysical studies of binocular contrast interaction (under non-rivalrous conditions) show that eye of presentation influences visual sensitivity at different locations across the visual field [20,21,22].

An asymmetry between nasal and temporal hemifields has been shown in ongoing rivalry dynamics, though with seemingly conflicting results. Fahle et al. (1987) found that targets in the temporal hemifield had a higher average dominance, but only in the far periphery (over 20° eccentricity) [20], while Chen and He (2003) reported higher average dominance in the nasal hemifields between 1° and 5° [14]. At the onset of rivalry, small binocular differences in rivalry may be especially apparent. In this initial rivalry stage, dominance appears to be more sensitive to stimulus imbalances, such as luminance differences [10], and areas of monocular dominance [4,8]. At the onset of rivalry, evidence of hemifield dominance has been shown close to the fovea, both with full color fields [23] and with discrete targets [4,8]. Leat and Woodhouse (1984) found evidence along the horizontal meridian for dominance patterns organized by hemifield using targets presented between 1° and 4° from the fovea. They report that even one degree of eccentricity is peripheral enough to estimate the hemifield pattern for the rest of an individual’s visual field up to 4° [8]. Such studies most frequently report temporal hemifield dominance [4,23], although considerable intersubject variation has also been shown [3,4,8,14], and not all studies show visible hemifield effects in individual data [7,9].

Within the fovea itself, predictions about general patterns of dominance must consider that the fovea possesses several properties that distinguish it from the surrounding retina. The fovea subtends about two degrees of visual angle and has a much higher cone density, no rods, and a smaller receptive field size [18]. With respect to rivalry dynamics, there are hints in the literature that they may behave differently at the fovea, with a single subjective report of rivalry showing higher switch rates in the fovea than in the periphery [24] and slower wave propagation [25]. It is possible that the various factors that influence onset rivalry in the periphery are absent or are overshadowed by other factors in the fovea, resulting in a pattern that differs from that seen across the wider visual field. To more accurately determine the nature of onset dominance near and around the fovea, we decided to use a sequence of small, overlapping grating patches to systematically sample the central 3° of the visual field. We aimed to determine if there are discernible patterns relating to eye dominance in this area, and in particular if hemifield effects seen in the periphery are also evident in the fovea and immediate surrounds. We were specifically interested in determining if radial patterns of onset bias are invariant with eccentricity, or whether the pattern of dominance at onset is more variable and patchwork, or even homogenous, across central vision. Onset effects will also be compared to continuous rivalry by using both 1-s and 1-min presentations.

In addition to the investigation of complete dominance, previous studies of binocular rivalry report a higher likelihood of mixed dominance in the fovea [26]. Consistent with this finding, early piloting in the current study revealed that participants had difficulty reporting a clear sense of dominance at onset for individual grating stimuli subtending 1° to 2° when they were presented in the fovea. Instead, observers reported a mixed percept that was nonetheless stable across presentations in its pattern of the grid or isolated patches corresponding to the two gratings. While it is this stable pattern that we hope to investigate, mixed percepts are common even with small targets. Because mixed percepts have such a high likelihood in the fovea, an understanding of dominance effects in this area must include an examination of the mixed report as well. Indeed, recent studies have suggested that the mixed percept is itself worthy of investigation, with this phase of rivalry being highlighted as being uniquely altered within clinical populations [27,28,29,30] and correlated with certain personality traits [31]. In the current study, therefore, we will track such percepts and include them in an understanding of onset dominance in rivalry.

## 2. Materials and Methods

### 2.1. Participants

Twelve experienced psychophysical observers participated in the study (including authors OC (S6), and JS (S7) and ten who were naïve to the research aims). However, three observers were excluded related to fixation testing, leaving 9 observers (6 females and 3 males) remaining in the final analysis. All observers had normal or corrected to normal vision. Participants were screened and consented in accordance with human subject protocols at the University of Melbourne.

### 2.2. Stimulus and Apparatus

**Figure 1:**
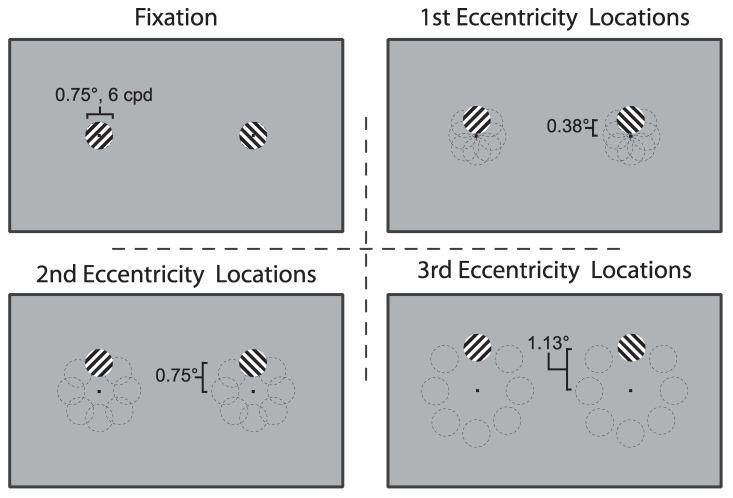
Stimuli Locations: The fovea was sampled in 25 overlapping locations, at fixation and at 8 locations each at 3 eccentricities. The thin dashed lines were not presented during the experiment but are shown here for clarity. To aid convergence during each presentation, the rivalry stimuli were presented within a single noise annulus (0.5° thick) framing the area of interest (not shown).

Stimuli were created using a MacBook Air (11-inch, Mid 2012; Apple, Cupertino, CA, USA) and a Dell OptiPlex 9020 computer (Dell, Round Rock, TX, USA) running Ubuntu 12.04.5 LTS, and all arrays were generated and displayed using Psychtoolbox and Eyelink Toolbox extensions in MATLAB R2013b [32,33]. Stimuli were presented with a 21” Sony CPD G520 CRT monitor with a vertical refresh rate of 100Hz. Orthogonal, oblique gratings were presented on a gray background (25.1 cd/m2) and viewed dichoptically through a mirror stereoscope from a distance of 57 cm for four initial observers, and 70 cm for the remaining eight (visual angle was consistent). The gratings were presented at a total of 25 locations: 1 at fixation and at 8 locations arranged in an imaginary circle surrounding fixation, centered at three eccentricities—0.38°, 0.75°, and 1.13°—from fixation (Figure 1). All patches subtended 0.75° with an achromatic sine wave grating of 6 cycles per degree. The locations surrounding fixation were overlapping and overall tiled an area subtending 3°. A small black circle served as a fixation point for surrounding gratings, including the foveal presentation. Gratings were presented within a ring of black and white noise to aid convergence. The noise annulus had a radius of 3.25° to the middle of the ring and was 0.5° thick.

### 2.3. Procedure

#### 2.3.1. Onset Rivalry

Gratings were presented for 1 s in a given location with a 9 s interstimulus interval. The long interstimulus interval was to minimize perceptual memory effects that can result from intermittent presentation in the same location [34]. The annulus and fixation points were present continuously throughout both the interstimulus period and during stimulus presentation followed by a 1-s blank screen. During the interstimulus period, the fixation point was white, changing to black 3 s before the grating was presented to signal to the observer to fixate and orient their attention to the task. Gratings were presented in each of the locations in pseudo-randomized order and were never presented in the same location on successive trials. Orientations were pseudo-randomized between eyes, with each orientation/eye combination occurring once each block, for a total of 10 presentations for each unique condition of eye, orientation, and location. Each location was sampled twice in each block, with a total of 10 blocks. Observers were asked to report their perception with a single key press using three separate keys, indicating a perception of left-tilting, right-tilting, or mixed percept. The criterion given to observers to report full dominance was around 85%. The duration of the onset rivalry testing was approximately 10 min per block.

#### 2.3.2. Sustained Rivalry

Stimulus gratings were identical to those in the onset task. Gratings were presented in each location for 1 min, and observers were asked to track dominance by holding down keys indicating a left-tilting grating or a right-tilting grating. To indicate mixed percept, an initial group of four observers (including S6 and S7) was asked to press both keys together, while the remaining eight observers instead held down a third key. Eye of presentation, orientation, and locations were pseudo-randomized across five blocks of testing. Each location was sampled twice—once with the left- and right-tilting gratings to the left and right eye, respectively, and again with the eye of presentation switched. After each 1-min presentation, observers were allowed to take a short break if desired. The total duration of the sustained rivalry testing was approximately 60 min, including breaks.

#### 2.3.3. Confirming Bias Stability

To assess the stability of onset dominance, we determined the net dominance for each location (Reye–Leye) and conducted a Pearson’s correlation comparing the first five blocks and the second five blocks of onset rivalry for each observer. All observers but one showed a significant correlation at *p* < 0.01. The average correlation coefficient (of those with a significant correlation) was r = 0.685 (range 0.518–0.866), *n* = 25, with average *p* = 0.002 (range <0.001–0.008). The only observer who did not show a significant correlation (S2; *r* = 0.336, *p* = 0.101) demonstrated a strong overall eye dominance, with little variability between locations.

#### 2.3.4. Confirming Fixation

To confirm that the observers were able to maintain fixation, eye tracking was employed in separate trials at the end of rivalry testing. One block of brief presentation mimicked one block of onset rivalry; however, no mirrors were used (the mirrors blocked the eye tracker), and stimuli were centrally and binocularly presented. Observers responded to an actually left- or right-tilting grating. A second block mimicked continuous rivalry and was also presented binocularly with gratings switching between left- and right-tilting every two seconds. During the continuous presentation block, gratings were presented only in the seven locations along the horizontal midline.

The spatial accuracy of the EyeLink 1000 (SR Research Ltd., Mississauga, Ontario, Canada) is 0.5 degrees [35]. Given the small separation of stimuli, we are unable to determine if saccades were made to stimuli at 0.38º eccentricity. However, we are able to determine two types of fixation failure. One is where participants followed the target, resulting in a strong correlation between eye position and stimulus location across trials, and one where there were large changes in fixation position that were not correlated with stimulus location (note that the latter may result from an inability to determine reliable eye position using the Eyelink, rather than an actual failure to fixate). Two observers were excluded using the above criteria for maintaining fixation, and whose standard deviation in fixation was larger than 1.5 times the distance from fixation to the center of the closest stimulus patch (approximately the spatial precision of the Eyelink). Another observer was not able to return for fixation trials and was excluded.

## 3. Results

### 3.1. Eye Dominance

**Figure 2:**
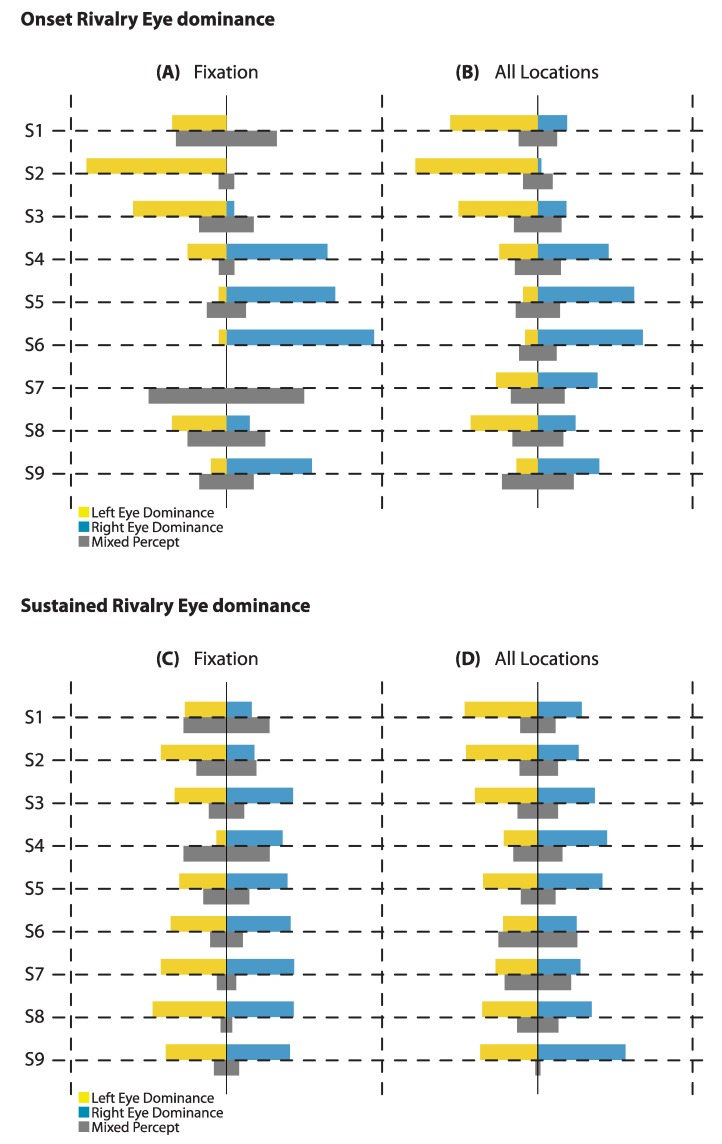
Eye Dominance: The proportion of right eye and left eye dominance at onset shows a stronger bias at fixation (**A**) than when averaging over all locations in and around the fovea (**B**). During sustained rivalry there is less bias, both at fixation (**C**) and over all locations (**D**), than at the onset of rivalry.

One aim of this study was to investigate any general effects of eye dominance in and around the fovea at the onset of rivalry. To assess this, we first examined bias at fixation, as well as the average bias across all locations (see Figure 2A,B). At onset, with a 1-s presentation time, the net proportion bias appeared larger for fixation than for the average proportions of all locations for most observers, suggesting the immediate surrounds are not homogenous with fixation. During sustained rivalry over 60 s of presentation, the bias at fixation and the average bias of all locations appear to be relatively similar for individuals (see Figure 2C,D).

To compare the extent of bias related to eye dominance at onset versus sustained rivalry, we did a paired T-test between the absolute value of the differences between the proportion of right eye dominance and the proportion of left eye dominance (|Reye–Leye|) at fixation. A paired-samples T-test revealed that the proportional strength of bias (of either eye) was stronger at onset (M = 0.489, SD = 0.300) than during sustained rivalry (M = 0.106, SD = 0.100); t(8) = 4.08, *p* = 0.004, after mixed responses were excluded. In all but one case, this was in the same direction, and demonstrates an attenuation of the bias when viewing rivalry over a sustained period. For one observer (S3), the bias during sustained rivalry was in the opposite direction to the bias at onset, but the bias was still not as strong.

### 3.2. Hemifield Dominance

#### 3.2.1. Onset Rivalry

**Figure 3:**
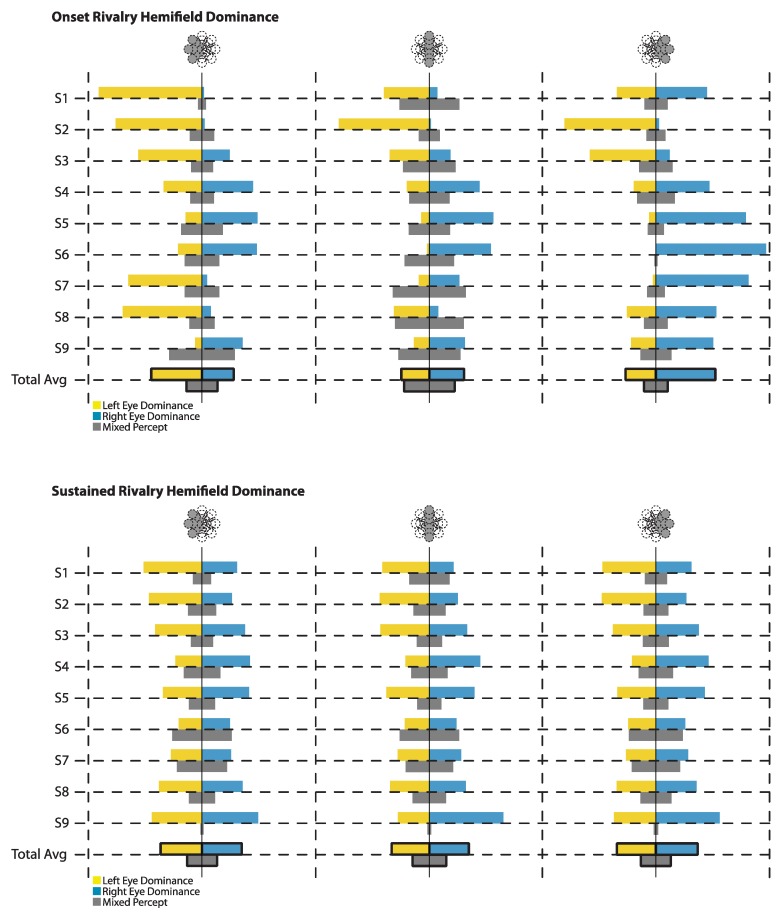
Hemifield Dominance: At the onset of rivalry, the net proportion of left eye dominance, right eye dominance, and mixed percept varies by region of the visual field. During sustained rivalry, left eye and right eye dominance still differ in the left and right hemifields, but mixed percept does not significantly differ across regions.

In addition to basic eye dominance, several observers show evidence of hemifield effects (Figure 3). To examine the extent of these effects across observers, we compared the net onset bias of the locations to the right and to the left of fixation. Bias for each observer was calculated as the difference in proportion (Reye–Leye; a negative proportion signifies a left eye bias) for both the left hemifield and the right hemifield. A paired T-test revealed a significant difference between the left hemifield (M = −0.239, SD = 0.497) and the right hemifield (M = 0.245, SD = 0.573), t(8) = −2.69, *p* = 0.028. With the left hemifield showing a higher dominance of the left eye (negative net proportion) and the right hemifield showing a higher dominance of the right eye (positive net proportion).

If hemifield effects are a factor in onset bias, one would also expect to see more mixed percept at the vertical midline, due to the competing influences of the hemifields meeting at this point. We used a one-way repeated measures ANOVA to compare the proportion of mixed percept of the left hemifield, midline, and right hemifield locations, which revealed a significant difference between these regions, F(2,16) = 10.367, *p* = 0.001. Post hoc tests showed the proportion of mixed percept at the midline was significantly more than in the left hemifield (*p* = 0.018) or the right hemifield (*p* = 0.002). The proportion of mixed percept did not differ between the left and the right hemifield (*p* = 0.238).

#### 3.2.2. Sustained Rivalry

When the rivalry stimuli were viewed continually for 60 s, the biases in the left hemifield (M = −0.015, SD = 0.127) and the right hemifield (M = 0.025, SD = 0.147) were not as pronounced as at the onset of rivalry, but were still significantly different, t(8) = -2.51, *p* = 0.036, with observers reporting more left eye dominance, on average, in the left hemifield, and more right eye dominance in the right hemifield.

Reported mixed percept was again compared for the left hemifield, midline, and right hemifield using a repeated measures ANOVA. Unlike at onset, however, there was no significant difference in the average mixed report between the three regions during sustained rivalry, F(2,16) = 2.124, *p* = 0.152.

### 3.3. Mixed Percept and Eccentricity

#### 3.3.1. Onset Rivalry

**Figure 4:**
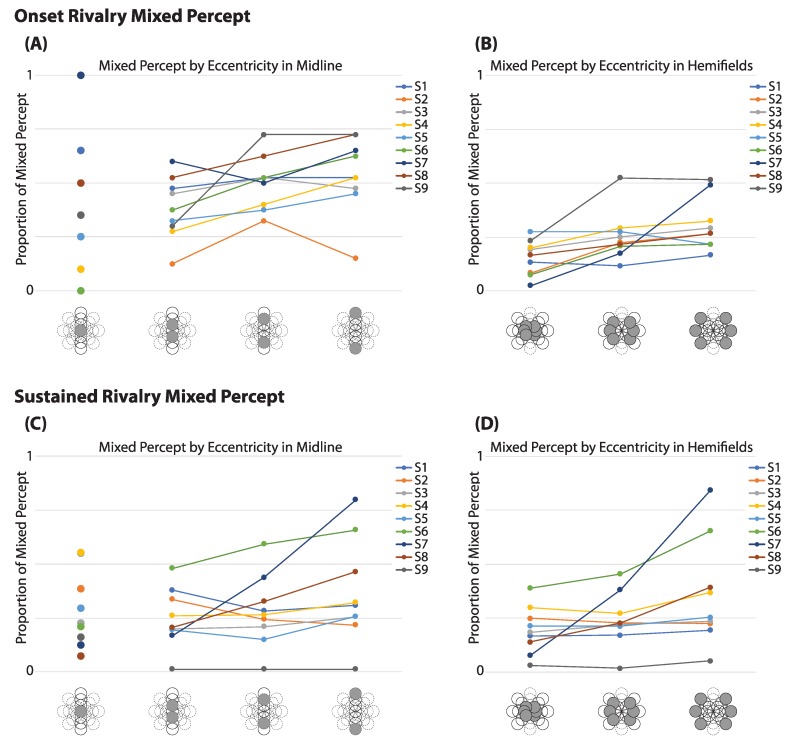
Mixed Report Along Eccentricities: The average proportion of mixed percept during onset at fixation and the three subsequent eccentricities along the midline (**A**) and in the hemifields (**B**). At onset, the proportion of mixed percept is highly variable between observers at fixation, but more closely clustered in the hemifields. During sustained rivalry, the proportions appear less spread out at fixation (shown with midline eccentricities (**C**)) and more spread out in the hemifields (**D**) than at rivalry onset.

In addition to an increase in mixed percept at the midline at onset, we also investigated whether mixed percept was affected by eccentricity from fixation. Because the increased mixed report at the vertical midline suggests the involvement of hemifield effects, which could interact with an evaluation of the effect of eccentricity, these locations, including fixation, were removed before analysis. Across observers, the proportion of mixed report at fixation spanned the full range from 0% to 100% (Figure 4A). At locations peripheral to fixation, however, there was a much smaller range in the proportions of mixed report among observers, and these appeared to increase on average with greater eccentricity (see Figure 4B). A repeated measures ANOVA of the lateral locations shows a significant effect of eccentricity in the report of mixed percept at onset, F(2, 16) = 7.65, (*p* = 0.005). (If midline locations, including fixation, are incorporated into the analysis, a repeated measures ANOVA with a Greenhouse–Geisser correction showed no significant effect of eccentricity, *F*(1.23, 9.82) = 1.75, *p* = 0.220.)

#### 3.3.2. Sustained Rivalry:

During sustained rivalry, the range itself of average mixed percept at fixation is not as visibly different from other eccentricities (Figure 4C). A repeated measures ANOVA with Greenhouse–Geisser correction showed no difference between eccentricities when the midline was removed, *F*(1.02, 8.14) = 4.04, *p* = 0.078 (Figure 4D). (When all locations were included, the repeated measures ANOVA with Greenhouse–Geisser correction was also not significant, *F*(1.23, 9.87) = 1.54, *p* = 0.250.)

### 3.4. Individual Onset Rivalry Response Profiles

**Figure 5:**
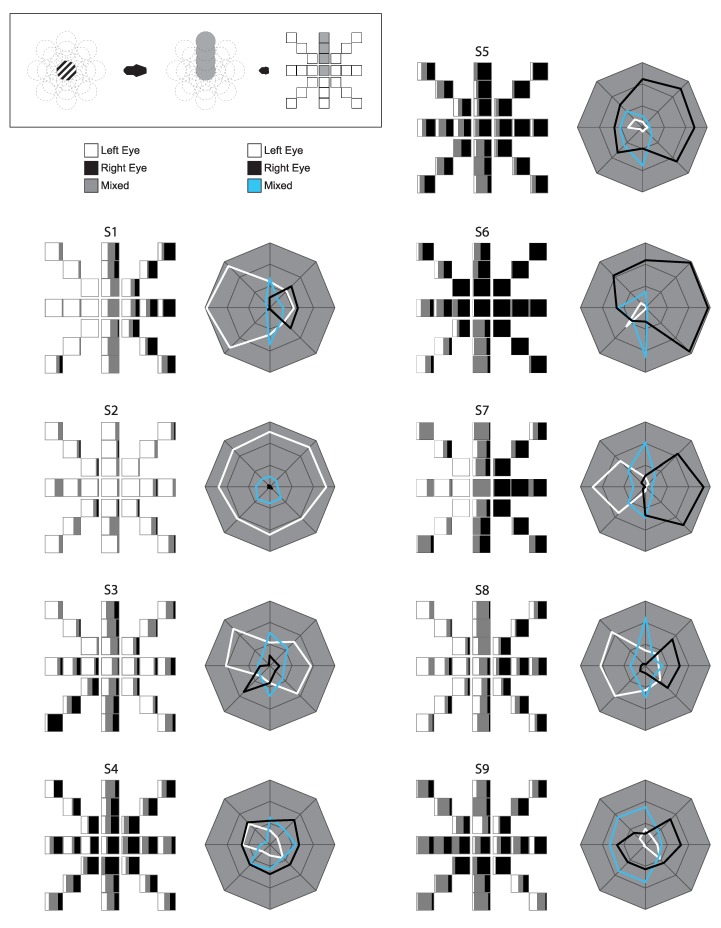
Individual Onset Rivalry Profiles: Raw data for individual observers. Boxes on the left represent each location tested, though spread out from their overlapping positions. Polar plots on the right represent each coordinate direction, but with eccentricities collapsed (and fixation omitted). Distance from the center indicates the average proportion of Left Eye, Right Eye, and Mixed Percept, respectively, across the three eccentricities at each coordinate.

Inspection of the raw, individual data suggests that the onset bias in the foveal range is neither homogenous nor completely random (Figure 5). Biases appear to have radial consistency, with similar biases seen along coordinates from central fixation outwards. Between-subject differences are evident, and both whole eye dominance and hemifield dominance appear to have varying degrees of influence on individuals’ patterns.

## 4. Discussion

The current study showed distinct patterns of onset dominance bias across different locations in the visual field, in agreement with previous work [3,4,7,9]. That these onset dominance patterns were seen for stimuli near the fixation point shows that even within the foveal range, eye dominance is not homogenous, and stable, idiosyncratic patterns of bias can be detected. These patterns appear to be influenced by temporal hemifield effects, though individual observers show considerable variation between their onset bias patterns.

At fixation, while the dominance biases were significantly greater at onset, biases towards the same eye were seen across onset and sustained rivalry, in all but one case. This result is in line with other studies that have shown a correlation between onset and sustained, at least for certain factors [7,9], while also demonstrating the onset stage of rivalry to be more sensitive to signal strength imbalances due to monocular dominance, as also seen with other low-level factors [10,12,36].

When responses were divided by hemifield, there was evidence of a temporal hemifield advantage, in line with some previous research done both in rivalry and other psychophysical measures [4,16,17,23]. The present result is perhaps surprising, however, as some of these previous studies showed hemifield differences primarily in the far periphery, with effects disappearing as stimulus presentation moves closer to central vision [16,17,20]. In the current study, by contrast, some observers show hemifield effects in locations that are in fact directly adjacent to fixation, as can be seen in the individual profiles (Figure 5). In sustained rivalry, the difference between hemifields was also significant, though the effect was smaller and there was no significant increase in mixed percept at the midline. Our data indicated a temporal hemifield advantage in ongoing rivalry, in contrast to Chen and He (2003) [14]. These differences perhaps speak to the considerable variation between individuals in the patterns of dominance across the visual field. Our data do show, however, that central vision is not exempt from these visual field influences, though the effect is apparently one of many factors determining dominance at the onset of rivalry.

When examining mixed report at onset, individual data suggested that mixed report increased with eccentricity for locations away from the fixation point, but showed much more variability between observers at fixation itself (Figure 4A,B). And in fact, when the vertical midline was removed to avoid interaction with hemifield effects, the increase in mixed report with eccentricity was significant but was not significant when the midline locations were included. To interpret this finding, it is worth noting that the mixed percept response in this study could include the perception of a superimposed grid, a patchwork of orientations, or even a herringbone-like percept (with perception of both gratings divided directly down the middle of the patch). It is possible that these different mixed percepts result from separate underlying mechanisms. For example, a patchy mixed percept could result from smaller zones of monocular dominance, as has been reported in the fovea [26], a ‘herringbone’-like percept from adjacent hemifields, or grid-like pattern from reduced visual acuity, as one might expect when gratings are presented in the periphery. Further research is needed to tease apart those percepts that cannot be reported as pure dominance. During sustained rivalry, eccentricity did not have a significant effect on the proportion of mixed percept, which could suggest that during ongoing switching, mixed percepts are dictated by other mechanisms unrelated to monocular dominance and visual field location. Such mechanisms could be more related to stimulus factors, such as target size and spatial frequency, as shown in O’Shea et al. (1997) [37]. It is still possible, however, that the visual qualities of the mixed percepts reported during ongoing switching (i.e., patchy, herringbone, or grid-like) are distinct based on location, and an experiment that was able to differentiate between such percepts would help delineate the nature of mixed percepts.

The current study suggests that factors related to visual field location can influence dominance at the onset of rivalry even into the foveal range. For example, such predominance appears to be influenced in part by naso/temporal hemifield asymmetries even close to fixation. While onset rivalry is more heavily biased by monocular dominance in general, the current study also shows that any imbalance during sustained rivalry is typically in the same eye as the bias at onset. This result suggests that monocular dominance plays a role in rivalry both at onset and during sustained stimulus viewing, but to a different degree. The extent of individual differences observed in the current study suggests that many factors are at play, and therefore, perceptual biases at onset and during sustained rivalry are unlikely to be determined by the same anatomical and/or physiological properties across populations. For individual observers, however, there appear to be consistent patterns of onset dominance, even within the foveal range, that are dependent on visual field location and eye of presentation. Therefore, basic visual imbalances between the eyes need to be taken into account in models of rivalry, particularly those models intended to be a general model of binocular interactions.
